# High-resolution fingerprinting of *Candida parapsilosis* isolates suggests persistence and transmission of infections among neonatal intensive care unit patients in Kuwait

**DOI:** 10.1038/s41598-018-37855-2

**Published:** 2019-02-04

**Authors:** Mohammad Asadzadeh, Suhail Ahmad, Noura Al-Sweih, Ferry Hagen, Jacques F. Meis, Ziauddin Khan

**Affiliations:** 1 Department of Microbiology, Faculty of Medicine, Kuwait University, Jabriya, Kuwait; 2 Department of Medical Mycology, Westerdijk Fungal Biodiversity Institute, Utrecht, Netherlands; 3 Department of Medical Microbiology and Infectious Diseases, Canisius Wilhelmina Hospital (CWZ), Nijmegen, Netherlands; 4 Centre of Expertise in Mycology, Radboudumc/CWZ, Nijmegen, Netherlands

## Abstract

*Candida parapsilosis* causes ~35% of all candidemia cases in neonates. High-resolution fingerprinting of *C*. *parapsilosis* isolates from neonatal intensive care unit (NICU) patients in Maternity Hospital (MH) was performed to identify epidemiologically related strains. Sixty-eight bloodstream/colonizing strains isolated from 59 NICU patients, two isolates from health care workers (HCWs) from MH and 18 bloodstream isolates from two other hospitals were used. Six microsatellite markers were employed, isolates were assigned a numerical microsatellite genotype (MSG), dendrogram was constructed and similarities between genotypes were visualized by minimum spanning tree. Fifty bloodstream isolates from MH yielded 37 MSGs with 20 isolates clustering in 7 MSGs. Duplicate isolates and colonizing strains yielded same/highly similar MSG as bloodstream isolates. Colonizing strains from two non-candidemia patients yielded unique MSGs while others belonged to a cluster. All isolates from HCWs and from two other hospitals belonged to unique MSGs. Cluster isolates came from patients in NICU-1 or from neonates in NICU-1 and other NICUs. Clonal complexes comprising closely related genotypes indicative of microevolution were also detected. Our data show that some *C*. *parapsilosis* strains have persisted in MH environment over several years and these endemic genotypes were transmitted to other patients in NICU-1 and/or other nearby NICUs.

## Introduction

*Candida* spp. colonize human skin, mucosal membranes and the gastrointestinal tract soon after birth^1,2^. They also cause opportunistic infections in immunocompromised and hospitalized patients^3^. *Candida* spp. are also among the four most common cause of late onset septicemia in very-low-birth-weight infants^4,5^. Although *Candida albicans* is the most common cause of candidemia/invasive candidiasis, nearly 50% of all *Candida* infections are now caused by other non-*albicans* species of *Candida*^6,7^. *Candida parapsilosis* is commonly associated with invasive candidiasis and in some centers/geographical areas it has even surpassed *C*. *albicans* in frequency^6–9^. This species is also commonly isolated from neonatal intensive care units (NICUs), causing ~35% of all candidemia cases in neonates^9–12^. *Candida parapsilosis* now comprises three closely-related species and most invasive infections are caused by *C*. *parapsilosis sensu stricto* isolates^13–15^. Although the main reservoir of *C*. *parapsilosis* in hospital environment remains unknown, nosocomial infections are facilitated by the spread of the yeast from the hands of health care workers (HCWs) or contaminated patient care equipment to susceptible patients^9–12,16,17^. This organism also has affinity for glucose-containing parenteral nutrition and forms biofilms on intravascular devices which also promote its spread in hospital settings^9–12^.

The source of invasive *C*. *parapsilosis* infections are investigated by molecular fingerprinting studies^9,16–20^. Recent studies utilizing multi-locus microsatellite typing have shown that *C*. *parapsilosis* infections mostly occur as a result of exogenous transmission of the organism to the patient, particularly from the hands of HCWs and have reported the presence of endemic genotypes in some settings^9,16,19–21^. However, these studies have mostly been carried out with adult patient populations^9,19–21^. Although studies involving neonates, including low/very-low-birth-weight neonates, have also been carried out, the number of isolates analyzed from a single hospital in most of these investigations were relatively small (<30)^9,11,17,18,22^. A total of 152 neonates were treated for culture-confirmed candidemia in various NICUs of Maternity Hospital (MH) in Kuwait during January 2010 to August 2014 and *C*. *parapsilosis* was the most common (61 of 152, 40%) *Candida* spp. isolated from bloodstream specimens of neonates. This study performed high-resolution fingerprinting of 70 bloodstream and colonizing *C*. *parapsilosis* isolates from NICU patients and HCWs in MH in Kuwait to identify epidemiologically related strains.

## Results

### Candidemia cases at Maternity Hospital and phenotypic and molecular characterization of study isolates

The MH in Kuwait is the main government hospital for the care of women during pregnancy and childbirth. Nearly 11,000 deliveries (corresponding to ~70% of all deliveries in Kuwait) are performed every year at MH. The MH has four NICUs (including special care units) which are in close proximity to each other. Premature very-low-birth-weight neonates are treated in NICU-1, low-birth-weight neonates are treated in NICU-2 while other neonates are treated in NICU-3 and NICU-4. The HCWs are assigned to their respective NICUs, however, this is not strictly observed. A total of 152 neonates were treated for culture-confirmed candidemia in various NICUs of MH in Kuwait during the study period of January 2010 to August 2014. Although *C*. *albicans* was the most common species during 2010 and 2011, *C*. *parapsilosis* surpassed *C*. *albicans* as the most common species causing candidemia in MH in Kuwait during January 2012 to August 2014.

A total of 61 neonates with culture confirmed candidemia due to *C*. *parapsilosis* were treated in the four NICUs during the study period. All patients were low/very-low-birth-weight (0.8 to 1.5 kg) preterm neonates on parenteral nutrition. Fifty *C*. *parapsilosis* bloodstream isolates from 50 neonates (representing 82% of all bloodstream isolates at MH during the study period) were available for fingerprinting studies. Of the 50 neonates, 34, five, five and six were treated in NICU-1, NICU-2, NICU-3 and NICU-4, respectively. Two duplicate bloodstream isolates from two neonates (collected within twelve days of isolation of the first isolate due to persistence of candidemia), six colonizing strains from other body sites of five neonates with candidemia, 10 colonizing strains from body sites of nine non-candidemia NICU patients and two surveillance cultures from hands of two HCWs attending neonates in NICUs were also used. Duplicate isolates were used to confirm whether the persistence of candidemia was due to the same or a different *C*. *parapsilosis* strain. Colonizing strains from candidemia patients, non-candidemia NICU patients and isolates from hands of two HCWs attending neonates in NICUs were used to see their relationship with strains causing candidemia. Additionally, nine randomly selected bloodstream isolates from eight adult and one pediatric candidemia patient (HA1 to HA9 cultured in Hospital A located within 1 km from MH) and representing 43% of all bloodstream *C*. *parapsilosis* from Hospital A during the study period were also used. Similarly, nine randomly selected bloodstream isolates from eight adult and one pediatric candidemia patient (HB1 to HB9 cultured in Hospital B located 17 km from MH) and representing 35% of all bloodstream *C*. *parapsilosis* from Hospital B during the study period were also used. The isolates from Hospital A and Hospital B were used for comparison purpose and to ascertain the discriminatory power of the fingerprinting technique employed in this study (Supplementary Table 1).

All 88 isolates were initially identified at the respective hospital microbiology laboratory as *C*. *parapsilosis sensu lato* by Vitek 2 (Biomerieux, Marcy l’Etoile, France). The multiplex PCR assay identified all 88 isolates as *C*. *parapsilosis sensu stricto*. The identification of selected isolates was further confirmed by species-specific PCR amplification of rDNA and/or by PCR-sequencing of ITS region of rDNA.

### Antifungal Susceptibility Testing

All 88 *C*. *parapsilosis* isolates were susceptible to amphotericin B, fluconazole, and caspofungin according to the CLSI breakpoints for *C*. *parapsilosis*^23^. The range of minimum inhibitory concentration (MIC) values of 88 *C*. *parapsilosis* isolates are summarized in Supplementary Table 2. The MIC_50_ and MIC_90_ values are also provided in Supplementary Table 2.

### Fingerprinting of *C*. *parapsilosis* isolates

**Figure 1:**
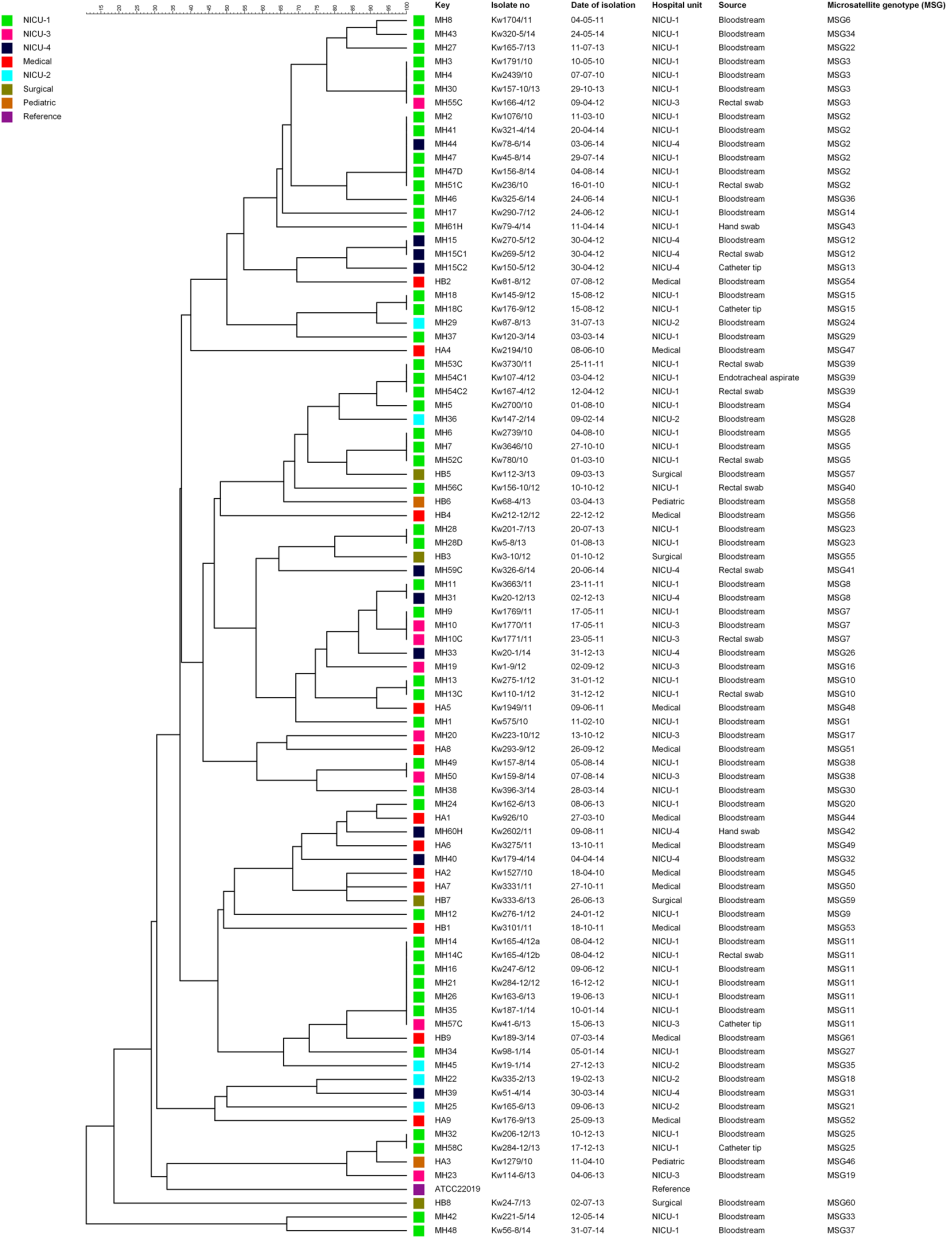
An UPGMA-derived dendrogram based on microsatellite fragments from 88 *C*. *parapsilosis* isolates. Similarity is presented in percentages using the scale bar in the upper left corner. The columns after the patient number refer to isolate number, date of isolation, hospital unit, source of the isolates and microsatellite-based genotype (MSG).

All 18 *C*. *parapsilosis* strains isolated from 18 candidemia patients at Hospital A and Hospital B analyzed in this study exhibited 18 unique microsatellite genotypes (MSGs) (Fig. 1, Supplementary Table 1). These studies confirmed that the six microsatellite loci-based fingerprinting method used in this study yields highly discriminatory patterns as all 18 isolates belonged to 18 unique genotypes. On the other hand, fingerprinting of 50 bloodstream *C*. *parapsilosis* isolates from 50 neonates from MH yielded only 37 MSGs. Interestingly, 30 isolates yielded 30 unique MSGs while 20 isolates were clustered in 7 MSGs (Supplementary Table 1). The three largest clusters included MSG11 with five isolates, MSG2 with four isolates and MSG3 with three isolates. The remaining four clusters (MSG5, MSG7, MSG8 and MSG38) contained two isolates each (Supplementary Table 1).

Duplicate isolates yielded the same MSG as the first isolate from the same patient. Similarly, the five colonizing strains from five neonates also yielded the same MSG as the bloodstream isolates from these patients. Interestingly, a second colonizing strain from one patient (MH15C_2_) yielded a unique MSG (MSG13). However, MSG13 differed from MSG12 in only one marker. Of the 10 colonizing strains from nine non-candidemia neonates, two isolates belonged to two unique MSGs (MSG40 and MSG41), five isolates from five neonates exhibited the same genotype as the unique or cluster MSG exhibited by the bloodstream isolates while the three colonizing strains from two neonates belonged to a new cluster (MSG39). The two *C*. *parapsilosis* strains from the hands of two HCWs belonged to unique genotypes (Supplementary Table 1).

**Figure 2:**
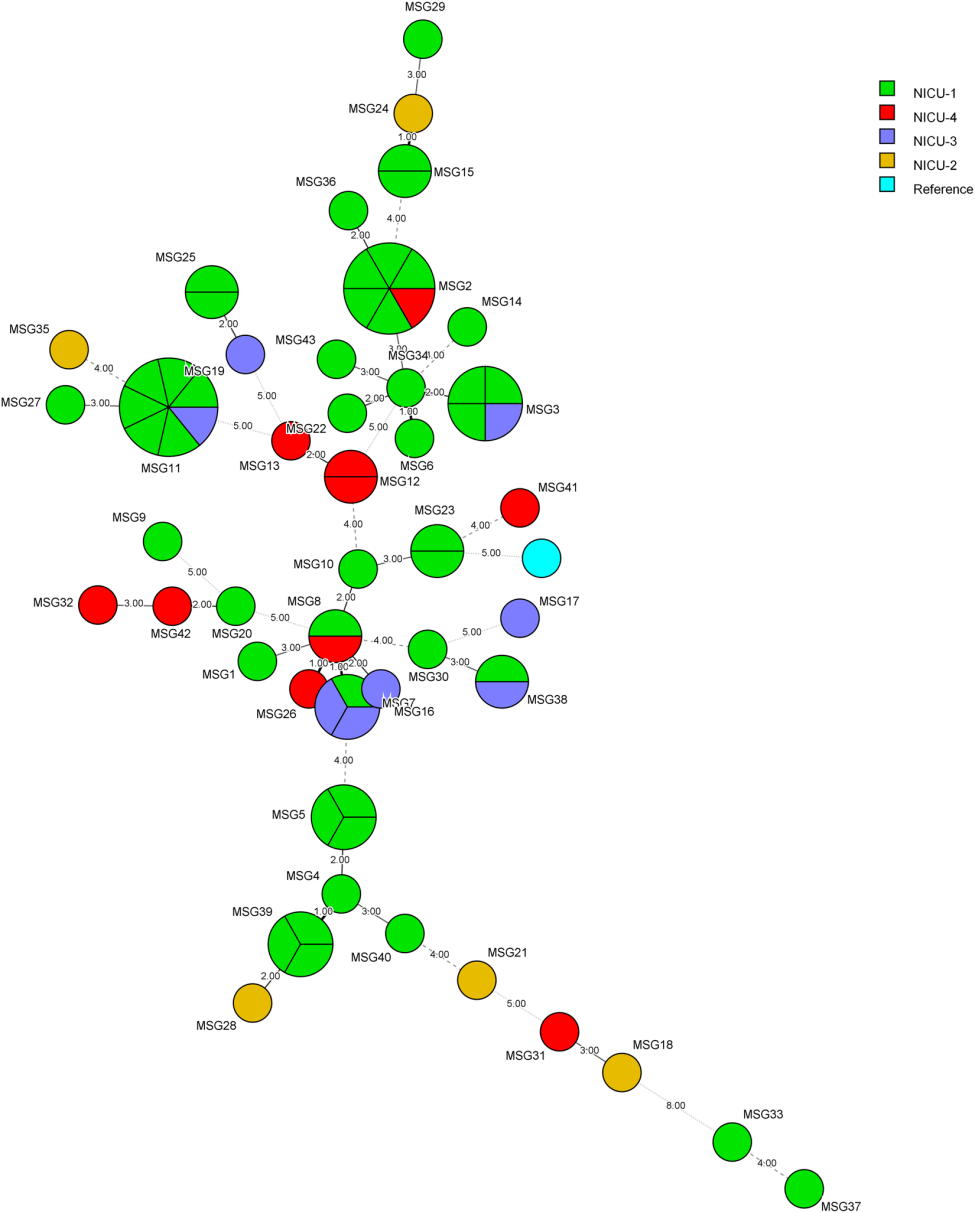
Minimum spanning tree of 70 *C*. *parapsilosis* isolates, from Maternity Hospital only, derived from microsatellite-based genotyping data. Each circle corresponds to a unique genotype, and lines between circles represent relative distance between isolates. The sizes of the circles correspond to the number of isolates in the same MSG. Connecting lines correspond to the number of differences between genotypes, with a solid thick line connecting genotypes that differ in one locus, a solid thin line connecting genotypes that differ in two or three loci, a dashed line connecting genotypes that differ in four loci, and a dotted line connecting genotypes that differ in more than four loci.

**Figure 3:**
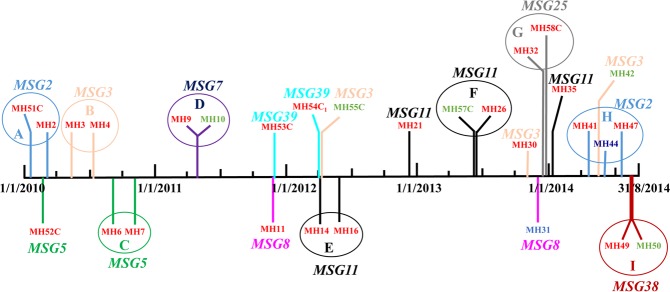
Color-coded timeline showing the distribution of 29 *C*. *parapsilosis* isolates exhibiting cluster fingerprinting patterns obtained from 29 patients in NICUs of the Maternity Hospital in Kuwait. The patient number (MH1, MH2, MH3 etc.) yielding *C*. *parapsilosis* at the indicated time points (vertical colored lines) are shown along with the corresponding cluster microsatellite genotype (MSG) in bold and italicized letters of same color. The location of patients in the four NICUs is indicated by patient number color: red font, patients in NICU-1; green font, patients in NICU-3 and blue font; patients in NICU-4. Colonizing strains are indicated by letter “C” after patient number. The patients yielding isolates belonging to same MSG that were recovered within 62 days of each other are shown in circles (color coded with each MSG) marked ‘A’ to ‘I’.

Overall, fingerprinting of 88 *C*. *parapsilosis* isolates from Kuwait identified 61 MSGs (MSG1 to MSG61; Fig. 1, Supplementary Table 1). Interestingly, 29 isolates from 29 candidemia and non-candidemia patients from MH clustered into 9 MSGs (excluding duplicate bloodstream isolates and colonizing isolates from the same candidemia patients which exhibited identical MSGs) (Fig. 1). The minimum spanning tree showed that all cluster isolates were obtained either exclusively from NICU-1 patients (isolates in MSG5, MSG25 and MSG39 clusters) or included isolates mostly collected from NICU-1 in addition to few isolates from other NICUs (isolates in MSG2, MSG3, MSG7, MSG8, MSG11, and MSG38 clusters) (Fig. 2). The data also showed that four clonal complexes (designated A to D) existed among *C*. *parapsilosis* isolates from MH which included closely related genotypes that differed in only one of the six microsatellite markers (Table 1). The color-coded time-line of isolation of cluster isolates obtained from different patients is presented in Fig. 3. An arbitrary window period of ≤62 days was used to define epidemiologically related isolates with identical MSG collected from two or more patients from the same or nearby NICU within MH. Based on this criteria, all isolates from neonates in NICU-1 for MSG2 (isolates from MH2 and MH51 patients in circle A), MSG3 (isolates from MH3 and MH4 patients in circle B), MSG5 (isolates from MH6 and MH7 patients in circle C), MSG11 (isolates from MH14 and MH16 patients in circle E) and MSG25 (isolates from MH32 and MH58 patients in circle G) were considered as epidemiologically related strains. Since HCWs are not strictly confined to their respective NICU, cluster isolates from neonates in different NICUs for MSG7 (isolates from MH9 and MH10 patients in circle D), MSG11 (isolates from MH26 and MH57 patients in circle F), MSG2 (isolates from MH41, MH44 and MH47 patients in circle H) and MSG38 (isolates from MH49 and MH50 patients in circle I) may also be considered as epidemiologically related strains. Furthermore, some genotypes (such as MSG3 and, to some extent, MSG8) were detected sporadically during a long period while others (MSG5, MSG7, MSG39, MSG25 and MSG38) were detected during a short period only. Interestingly, MSG11 persisted in the NICUs of MH for nearly two years (April 2012 to January 2014). On the other hand, MSG2, detected in early 2010, was not detected for four years (April 2010 to March 2014) until it re-appeared in April 2014 (Fig. 3).

**Table 1:** *Candida parapsilosis* genotypes identified from the Maternity Hospital and forming clonal complex.

Microsatellite genotype (MSG)	Patient no.^a^	Microsatellite fragment length-based allelic profile^b^												Clonal complex
		CanpaSTR3A/a	CanpaSTR3A/b	CanpaSTR3B/a	CanpaSTR3B/b	CanpaSTR3C/a	CanpaSTR3C/b	CanpaSTR6A/a	CanpaSTR6A/b	CanpaSTR6B/a	CanpaSTR6B/b	CanpaSTR6C/a	CanpaSTR6C/b	
MSG2	MH2, MH41, MH44, MH47, MH47D, MH51C	26	26	37	46	39	52	7	7	6	6	7	7	A
MSG36	MH46	26	26	37	46	39	52	7	7	**9**	**14**	7	7	
MSG3	MH3, MH4, MH30, MH55C	26	26	47	47	40	40	7	7	6	6	7	7	B
MSG34	MH43	26	26	47	47	**39**	**39**	7	7	6	6	7	7	
MSG7	MH9, MH10, MH10C	25	27	32	32	38	42	11	11	6	6	7	7	C
MSG8	MH11, MH31	25	27	32	32	**36**	42	11	11	6	6	7	7	
MSG1	MH1	25	25	32	32	**27**	**27**	11	11	6	6	7	7	
MSG12	MH15, MH15C_1_	26	32	32	32	44	44	7	7	6	6	7	7	D
MSG13	MH15C_2_	26	32	32	32	44	44	**12**	**12**	6	6	7	7	

^a^MH: patients from Maternity Hospital; MH51C: colonizing isolate from patient 51 at Maternity Hospital; MH47D: duplicate bloodstream isolate from patient 47 at Maternity Hospital. ^b^Bold numbers indicate marker differences within the clonal complex.

## Discussion

The origin of nosocomial *Candida* infections in adult patients could be colonizing endogenous strains or exogenous strains transmitted to the patients from biomedical devises, contaminated infusates, hospital personnel and environments^24–26^. Among preterm (low/very-low-birth-weight) neonates, early onset bloodstream infections are usually caused by organisms transmitted vertically from mother to infants during or shortly after delivery, however, late onset bloodstream infections may also be caused by organisms acquired during the course of hospital care^5,27^. *C*. *parapsilosis sensu stricto* isolates have a remarkable capability to easily colonize human skin (such as the hands of HCWs) and contaminate hospital surroundings^10,12^. Genotyping methods based on length variations associated with microsatellites have been developed for *C*. *parapsilosis* and are now considered as the most popular and versatile typing schemes due to their rapid evolution and ability to detect microevolutionary variations^28,29^. These methods, however, differ in the number of polymorphic microsatellite markers (varying from 4 to 6 loci) and consequently the discriminatory power of the typing scheme^11,17,28–30^. In this study, we have used the six-marker microsatellite panel which yields 12 alleles per strain, due to diploid nature of the organism, with maximum discriminatory power described so far^30^. Application of six-marker microsatellite panel yielded high-resolution fingerprinting of *C*. *parapsilosis* isolates in Kuwait. This is evident from the fact that all 18 randomly selected isolates collected from candidemia patients from two major hospitals belonged to different genotypes (MSG44 to MSG61).

When microsatellite genotyping was applied to 50 bloodstream isolates from neonates from MH, 30 isolates were identified as unique strains while 20 isolates were clustered in 7 MSGs. Duplicate isolates from two patients and colonizing strains from five neonates yielded the same genotype as the bloodstream isolate. However, a colonizing strain (MH15C_2_ from the catheter tip) from one patient yielded a different genotype than bloodstream and gastrointestinal tract isolates (MH15 and MH15C_1_) but differed in a single microsatellite marker only. Only few studies have analyzed multiple isolates from bloodstream and/or other body sites from the same patient. Multiple isolates from nine of 18 patients in one study yielded the same MSG, two patients yielded closely-related MSGs differing in only one marker while seven patients yielded unrelated MSGs from multiple sites^30^. Another study reported identical MSGs in serial isolates from the same or multiple anatomic sites of four of six patients while in the remaining two cases, the isolates differed in one or two markers^20^. Multiple isolates from two patients also yielded closely-related MSGs differing in only one marker in another study^11^. Taken together, these findings are consistent with spontaneous variation in microsatellite markers leading to microevolution within the same patient or in different patients infected with the same strain^9,30^.

Further analysis of 10 colonizing strains from nine non-candidemia patients admitted in the same NICUs showed that only two isolates belonged to a unique MSG (MSG40 and MSG41), three isolates from two neonates belonged to a new cluster (MSG39) while the remaining five isolates belonged to other genotypes prevalent in the same or adjoining NICUs. Thus a total of nine clusters comprising MSG2, MSG3, MSG5, MSG7, MSG8, MSG11, MSG25, MSG38 and MSG39 were identified. Interestingly all isolates in three clusters (comprising MSG5, MSG25 and MSG39) and nearly all isolates in the remaining six clusters (comprising MSG2, MSG3, MSG7, MSG8, MSG11 and MSG38) were obtained from neonates in NICU-1 which houses very-low-birth-weight neonates. Furthermore, some genotypes were observed during a short window period (MSG7, MSG25, MSG38 and to some extent MSG5 and MSG39) while others (MSG2, MSG3, MSG8 and MSG11) showed a more extended distribution spread over several years. In many cases (denoted by circles “A” to ‘I’ in Fig. 3), cluster isolates were obtained from patients admitted to NICUs within 62 days of each other, typically in the same NICU, suggesting persistence of some genotypes in MH in Kuwait. A window period of 60–90 days has also been used previously by other investigators for defining epidemiologically-linked cluster isolates of *Candida albicans* causing bloodstream infections^24,31^. Endemic genotypes have also been reported to persist within the hospital environment for several years causing nosocomial outbreaks of invasive *Candida* infections in several studies^18,25,32^. The isolation of clonally-related genotypes in both time and space supports nosocomial transmission of *C*. *parapsilosis* among some neonates in MH in Kuwait. Similar observations were reported from NICU patients in Italy and among neonates in one (Hospital B) of three hospitals from USA^11,18^. Although microbiological testing of HCWs or hospital environment was not carried out in these studies, it is probable that patient care and manipulation of catheters by HCWs was the source of *C*. *parapsilosis* in very-low-birth-weight neonates in NICU as most infants are not colonized by yeast species at birth^1,2,10,12^. The involvement of HCWs in cross-transmission of *C*. *parapsilosis* infection has been demonstrated in few studies^9,16,17^. One recent study has shown that *C*. *parapsilosis* isolates from the hands of most of the HCWs belonged to a unique genotype. However, genotypes of some *C*. *parapsilosis* isolates obtained from the hospital environment and the hands of HCWs were also common with bloodstream isolates obtained from NICU patients implicating the environment and the hands of HCWs as a cause of nosocomial infections^9^. However, the source of infection among our cluster isolates remained unknown as the two *C*. *parapsilosis* isolates recovered from the hands of two HCWs belonged to unique genotypes which were different from the cluster genotypes.

The presence of clonal complexes of closely related genotypes as a result of microevolution due to intrinsic instability of microsatellite loci has been observed in few studies^9,18,21,30^. However, many of these studies were based on analysis of only four microsatellite loci and the close association may have resulted from fortuitous grouping of the isolates due to lower discriminatory power of these typing schemes^9,18,21^. Our data based on analysis of six microsatellite loci also identified some genotypes in which minor changes had occurred in a single locus compared to the genotype of the cluster isolates. These closely related genotypes were obtained either from the same patient (MH15) or were found mainly among isolates from NICU-1 patients strongly supporting the possibility of microevolution from the parental strain^30^. The results of antifungal drug susceptibility testing showed that all 88 *C*. *parapsilosis* isolates were susceptible to amphotericin B, fluconazole and caspofungin and therefore, no association of antifungal resistance with specific genotypes was found. On the contrary, resistance to fluconazole was found in 77 of 143 (54%) *C*. *parapsilosis* isolates from NICU patients in South Africa and vast majority of isolates in four clusters comprised fluconazole-resistant strains^33^.

Our study has few limitations. i) Surveillance cultures from the mothers of neonates were not available. ii) The hands of the HCWs from the four NICUs in MH were not screened systematically and without prior notice which may have resulted in the recovery of *C*. *parapsilosis* from the hands of only two HCWs. iii) Environmental samples from the NICUs were not screened to trace an epidemiological link. iv) Clinical characteristics, major risk factors (other than very-low-birth-weight of the neonates) and the outcome of candidemia were not evaluated.

In conclusion, all 18 *C*. *parapsilosis* isolates from candidemia patients from two major hospitals belonged to different genotypes showing high discriminatory power of the six microsatellite marker-based fingerprinting method used in the present study. Our fingerprinting data from MH showed that 19 of 68 *C*. *parapsilosis* isolates from 19 preterm neonates in NICUs with/without candidemia (excluding duplicate bloodstream isolates and colonizing isolates from the same candidemia patients which exhibited identical MSGs) belonged to a cluster genotype. Furthermore, these cluster isolates were obtained from different patients within a short (62 days) time period. These findings suggest that some strains have persisted in the MH environment, particularly in NICU-1, over several years and these endemic genotypes were transmitted to other patients in NICU-1 and other nearby NICUs in MH in Kuwait. However, the source of infection among neonates remained unidentified as only two isolates were obtained from the hands of HCWs which belonged to different genotypes. This is the first report describing high-resolution fingerprinting of *C*. *parapsilosis sensu stricto* isolates from NICU patients from the Middle East.

## Materials and Methods

### Study setting and clinical isolates

This retrospective study was carried out on bloodstream *C*. *parapsilosis* strains isolated at Maternity Hospital (MH), Kuwait. A total of 70 *C*. *parapsilosis* strains isolated during January 2010 to August 2014 were analyzed. These included 50 *C*. *parapsilosis* bloodstream isolates from 50 neonates, two duplicate bloodstream isolates from two neonates (collected within twelve days of isolation of the first isolate), six colonizing strains from other (rectal swab, n = 4; catheter tip, n = 2) body sites of five neonates with candidemia, 10 colonizing strains from body sites (rectal swab, n = 7; catheter tip, n = 2 and endotracheal aspirate, n = 1) of nine non-candidemia NICU patients and two surveillance cultures from hands of two HCWs attending neonates in NICUs. Additionally, 18 bloodstream isolates from 16 adults and two pediatric candidemia patients from two other hospitals were also used for comparison purpose. The clinical specimens yielding *C*. *parapsilosis* were collected from neonates/patients after obtaining informed verbal consent from a parent and/or legal guardian (for patients under the age of 18 years old) as part of routine diagnostic work-up for the isolation of bacterial/fungal pathogens. The isolates were sent to the Mycology Reference Laboratory (MRL), Department of Microbiology, Faculty of Medicine, Kuwait University for identification and antifungal susceptibility testing and data on deidentified samples are reported in this study. *C*. *parapsilosis* (ATCC22019 = CBS604) and *C*. *albicans* (ATCC90028 = CBS8837) were used as reference strains. All isolates were subjected to species-specific identification by phenotypic and molecular methods, antifungal drug susceptibility testing and high-resolution fingerprinting by six loci-based multilocus microsatellite typing.

### Phenotypic and molecular identification

The isolates were initially identified as *C*. *parapsilosis sensu lato* at the original hospital by Vitek 2 yeast identification system (bioMérieux, Marcy-l’Etoile, France). All isolates were grown at MRL for 48 h on Sabouraud dextrose agar medium at 37 °C. The genomic DNA from the isolates was extracted by using the Gentra Puregene Yeast DNA extraction kit (Qiagen, Hilden, Germany) according to the instructions supplied by the manufacturer. Molecular identification was performed by a multiplex PCR assay that discriminates *C*. *parapsilosis sensu stricto* from *Candida orthopsilosis* and *Candida metapsilosis* among *C*. *parapsilosis sensu lato* strains, as described previously^32^. The identity of randomly selected isolates was further confirmed by *C*. *parapsilosis sensu stricto*-specific PCR assay described previously^14^ and DNA sequencing of internal transcribed spacer (ITS) region of rDNA by using panfungal ITS1 and ITS4 primers, as described previously^34,35^. The study was approved by the Joint Committee for the Protection of Human Subjects in Research, Health Sciences Center, Kuwait University and Ministry of Health, Kuwait and all the methods and investigations reported in this study were carried out according to their guidelines. The clinical samples yielding the isolates used in this study were taken after obtaining informed verbal consent of the patients as part of routine diagnostic work-up and this procedure was approved by the relevant ethical committees.

### Antifungal drug susceptibility testing

The susceptibility of *C*. *parapsilosis* isolates against antifungal drugs, amphotericin B, fluconazole and caspofungin was determined by Etest (bioMérieux, Marcy-L´Etoile, France) as described previously^36^. The revised interpretive susceptibility breakpoints as recommended by Clinical Laboratory Standards Institute (CLSI) were used for fluconazole (≤2 µg/ml, susceptible; 4 µg/ml, susceptible dose-dependent and >8 µg/ml, resistant) and caspofungin (≤2 µg/ml, susceptible; 4 µg/ml, intermediate and >8 µg/ml, resistant)^23^. Due to lack of defined breakpoints for amphotericin B, an isolate showing an MIC ≤ 1 µg/ml was considered as susceptible. Quality control was ensured by testing *C*. *parapsilosis* ATCC22019 (=CBS604) and *C*. *albicans* ATCC90028 (=CBS8837), as recommended by CLSI^23^.

### Fingerprinting by multilocus microsatellite typing

Fingerprinting of *C*. *parapsilosis* isolates was performed by using a panel of six short tandem repeat (STR) markers as described by Diab-Elschahawi *et al*.^30^. The alleles were designated by their sizes which were recorded by using GeneScan 500 LIZ size standard (Applied Biosystems) in the 35–500 nucleotide range. The number of repeats in each marker was determined by comparing the relative size of each allele to the reference *C*. *parapsilosis* strain CDC317 that has been subjected to whole genome sequencing (GenBank accession no. HE605206) and the combination of allele numbers for all six diploid loci was assigned an arbitrary microsatellite genotype (MSG). Based on the allele number for the six diploid loci for each isolate, a dendrogram was constructed by using BioNumerics v7.6 software (Applied Maths, Saint-Martens-Latem, Belgium) and standard unweighted pair group method with arithmetic mean (UPGMA) settings, as described previously^24^. The genetic relationship between the genotypes was studied by constructing a minimum spanning tree. The isolates from the same or different patients were considered belonging to the same MSG (cluster) when they possessed the same alleles for all six diploid loci. Closely related MSGs which differed in only one of six diploid microsatellite marker were grouped into clonal complexes. The reproducibility of the fingerprinting method was ensured by including the reference *C*. *parapsilosis* strain (ATCC22019 = CBS604) in each run.

## Supplementary information

Tables 1 and 2
