# Supplementary material for: High-resolution fingerprinting of Candida parapsilosis isolates suggests persistence and transmission of infections among neonatal intensive care unit patients in Kuwait
Source: Sci Rep. 2019 Feb 4;9:1340. doi: 10.1038/s41598-018-37855-2 (PMC6361955; doi:10.1038/s41598-018-37855-2)
Supplement: Supplementary file 1 — Tables 1 and 2 [file 41598_2018_37855_MOESM1_ESM.docx]

High-resolution fingerprinting of *Candida parapsilosis* isolates suggests persistence and transmission of infections among neonatal intensive care unit patients in Kuwait

Mohammad Asadzadeh^1^, Suhail Ahmad^1^, Noura Al-Sweih^1^, Ferry Hagen^2,3^, Jacques F. Meis^3,4^ and Ziauddin Khan^1*^

^1^Department of Microbiology, Faculty of Medicine, Kuwait University, Kuwait

^2^Department of Medical Mycology, Westerdijk Fungal Biodiversity Institute, Utrecht, Netherlands

^3^Department of Medical Microbiology and Infectious Diseases, Canisius Wilhelmina Hospital (CWZ), Nijmegen, Netherlands

^4^Centre of Expertise in Mycology, Radboudumc/CWZ, Nijmegen, Netherlands

*Corresponding author: Prof. Ziauddin Khan

**Supplementary Table 1:** Demographic and other details of patients and fingerprinting data for 88 *C. parapsilosis* isolates from Kuwait by microsatellite genotyping

| **Sno.** | **Patient no.^a^** | **Isolate no.** | **Date of isolation** | **Hospital unit** | **Source** | **Microsatellite genotype (MSG)** |
| --- | --- | --- | --- | --- | --- | --- |
| 1 | MH1 | Kw575/10 | 11-02-10 | NICU-1 | Bloodstream | MSG1 |
| 2 | MH2 | Kw1076/10 | 11-03-10 | NICU-1 | Bloodstream | MSG2 |
| 3 | MH3 | Kw1791/10 | 10-05-10 | NICU-1 | Bloodstream | MSG3 |
| 4 | MH4 | Kw2439/10 | 07-07-10 | NICU-1 | Bloodstream | MSG3 |
| 5 | MH5 | Kw2700/10 | 01-08-10 | NICU-1 | Bloodstream | MSG4 |
| 6 | MH6 | Kw2739/10 | 04-08-10 | NICU-1 | Bloodstream | MSG5 |
| 7 | MH7 | Kw3646/10 | 27-10-10 | NICU-1 | Bloodstream | MSG5 |
| 8 | MH8 | Kw1704/11 | 04-05-11 | NICU-1 | Bloodstream | MSG6 |
| 9 | MH9 | Kw1769/11 | 17-05-11 | NICU-1 | Bloodstream | MSG7 |
| 10 | **MH10** | Kw1770/11 | 17-05-11 | NICU-3 | Bloodstream | MSG7 |
| 11 | **MH10C** | Kw1771/11 | 23-05-11 | NICU-3 | Rectal swab | MSG7 |
| 12 | MH11 | Kw3663/11 | 23-11-11 | NICU-1 | Bloodstream | MSG8 |
| 13 | MH12 | Kw276-1/12 | 24-01-12 | NICU-1 | Bloodstream | MSG9 |
| 14 | **MH13** | Kw275-1/12 | 31-01-12 | NICU-1 | Bloodstream | MSG10 |
| 15 | **MH13C** | Kw110-1/12 | 31-12-12 | NICU-1 | Rectal swab | MSG10 |
| 16 | **MH14** | Kw165-4/12a | 08-04-12 | NICU-1 | Bloodstream | MSG11 |
| 17 | **MH14C** | Kw165-4/12b | 08-04-12 | NICU-1 | Rectal swab | MSG11 |
| 18 | **MH15** | Kw270-5/12 | 30-04-12 | NICU-4 | Bloodstream | MSG12 |
| 19 | **MH15C_1_** | Kw269-5/12 | 30-04-12 | NICU-4 | Rectal swab | MSG12 |
| 20 | **MH15C_2_** | Kw150-5/12 | 30-04-12 | NICU-4 | Catheter tip | MSG13 |
| 21 | MH16 | Kw247-6/12 | 09-06-12 | NICU-1 | Bloodstream | MSG11 |
| 22 | MH17 | Kw290-7/12 | 24-06-12 | NICU-1 | Bloodstream | MSG14 |
| 23 | **MH18** | Kw145-9/12 | 15-08-12 | NICU-1 | Bloodstream | MSG15 |
| 24 | **MH18C** | Kw176-9/12 | 15-08-12 | NICU-1 | Catheter tip | MSG15 |
| 25 | MH19 | Kw1-9/12 | 02-09-12 | NICU-3 | Bloodstream | MSG16 |
| 26 | MH20 | Kw223-10/12 | 13-10-12 | NICU-3 | Bloodstream | MSG17 |
| 27 | MH21 | Kw284-12/12 | 16-12-12 | NICU-1 | Bloodstream | MSG11 |
| 28 | MH22 | Kw335-2/13 | 19-02-13 | NICU-2 | Bloodstream | MSG18 |
| 29 | MH23 | Kw114-6/13 | 04-06-13 | NICU-3 | Bloodstream | MSG19 |
| 30 | MH24 | Kw162-6/13 | 08-06-13 | NICU-1 | Bloodstream | MSG20 |
| 31 | MH25 | Kw165-6/13 | 09-06-13 | NICU-2 | Bloodstream | MSG21 |
| 32 | MH26 | Kw163-6/13 | 19-06-13 | NICU-1 | Bloodstream | MSG11 |
| 33 | MH27 | Kw165-7/13 | 11-07-13 | NICU-1 | Bloodstream | MSG22 |
| 34 | **MH28** | Kw201-7/13 | 20-07-13 | NICU-1 | Bloodstream | MSG23 |
| 35 | **MH28D** | Kw5-8/13 | 01-08-13 | NICU-1 | Bloodstream | MSG23 |
| 36 | MH29 | Kw87-8/13 | 31-07-13 | NICU-2 | Bloodstream | MSG24 |
| 37 | MH30 | Kw157-10/13 | 29-10-13 | NICU-1 | Bloodstream | MSG3 |
| 38 | MH31 | Kw20-12/13 | 02-12-13 | NICU-4 | Bloodstream | MSG8 |
| 39 | MH32 | Kw206-12/13 | 10-12-13 | NICU-1 | Bloodstream | MSG25 |
| 40 | MH33 | Kw20-1/14 | 31-12-13 | NICU-4 | Bloodstream | MSG26 |
| 41 | MH34 | Kw98-1/14 | 05-01-14 | NICU-1 | Bloodstream | MSG27 |
| 42 | MH35 | Kw187-1/14 | 10-01-14 | NICU-1 | Bloodstream | MSG11 |
| 43 | MH36 | Kw147-2/14 | 09-02-14 | NICU-2 | Bloodstream | MSG28 |
| 44 | MH37 | Kw120-3/14 | 03-03-14 | NICU-1 | Bloodstream | MSG29 |
| 45 | MH38 | Kw396-3/14 | 28-03-14 | NICU-1 | Bloodstream | MSG30 |
| 46 | MH39 | Kw51-4/14 | 30-03-14 | NICU-4 | Bloodstream | MSG31 |
| 47 | MH40 | Kw179-4/14 | 04-04-14 | NICU-4 | Bloodstream | MSG32 |
| 48 | MH41 | Kw321-4/14 | 20-04-14 | NICU-1 | Bloodstream | MSG2 |
| 49 | MH42 | Kw221-5/14 | 12-05-14 | NICU-1 | Bloodstream | MSG33 |
| 50 | MH43 | Kw320-5/14 | 24-05-14 | NICU-1 | Bloodstream | MSG34 |
| 51 | MH44 | Kw78-6/14 | 03-06-14 | NICU-4 | Bloodstream | MSG2 |
| 52 | MH45 | Kw19-1/14 | 27-12-13 | NICU-2 | Bloodstream | MSG35 |
| 53 | MH46 | Kw325-6/14 | 24-06-14 | NICU-1 | Bloodstream | MSG36 |
| 54 | **MH47** | Kw45-8/14 | 29-07-14 | NICU-1 | Bloodstream | MSG2 |
| 55 | **MH47D** | Kw156-8/14 | 04-08-14 | NICU-1 | Bloodstream | MSG2 |
| 56 | MH48 | Kw56-8/14 | 31-07-14 | NICU-1 | Bloodstream | MSG37 |
| 57 | MH49 | Kw157-8/14 | 05-08-14 | NICU-1 | Bloodstream | MSG38 |
| 58 | MH50 | Kw159-8/14 | 07-08-14 | NICU-3 | Bloodstream | MSG38 |
| 59 | MH51C | Kw236/10 | 16-01-10 | NICU-1 | Rectal swab | MSG2 |
| 60 | MH52C | Kw780/10 | 01-03-10 | NICU-1 | Rectal swab | MSG5 |
| 61 | MH53C | Kw3730/11 | 25-11-11 | NICU-1 | Rectal swab | MSG39 |
| 62 | **MH54C_1_** | Kw107-4/12 | 03-04-12 | NICU-1 | Endotracheal aspirate | MSG39 |
| 63 | **MH54C_2_** | Kw167-4/12 | 12-04-12 | NICU-1 | Rectal swab | MSG39 |
| 64 | MH55C | Kw166-4/12 | 09-04-12 | NICU-3 | Rectal swab | MSG3 |
| 65 | MH56C | Kw156-10/12 | 10-10-12 | NICU-1 | Rectal swab | MSG40 |
| 66 | MH57C | Kw41-6/13 | 15-06-13 | NICU-3 | Catheter tip | MSG11 |
| 67 | MH58C | Kw284-12/13 | 17-12-13 | NICU-1 | Catheter tip | MSG25 |
| 68 | MH59C | Kw326-6/14 | 20-06-14 | NICU-4 | Rectal swab | MSG41 |
| 69 | MH60H | Kw2602/11 | 09-08-11 | NICU-4 | Hand swab | MSG42 |
| 70 | MH61H | Kw79-4/14 | 11-04-14 | NICU-1 | Hand swab | MSG43 |
| 71 | HA1 | Kw926/10 | 27-03-10 | Medical | Bloodstream | MSG44 |
| 72 | HA2 | Kw1527/10 | 18-04-10 | Medical | Bloodstream | MSG45 |
| 73 | HA3 | Kw1279/10 | 11-04-10 | Pediatric | Bloodstream | MSG46 |
| 74 | HA4 | Kw2194/10 | 08-06-10 | Medical | Bloodstream | MSG47 |
| 75 | HA5 | Kw1949/11 | 09-06-11 | Medical | Bloodstream | MSG48 |
| 76 | HA6 | Kw3275/11 | 13-10-11 | Medical | Bloodstream | MSG49 |
| 77 | HA7 | Kw3331/11 | 27-10-11 | Medical | Bloodstream | MSG50 |
| 78 | HA8 | Kw293-9/12 | 26-09-12 | Medical | Bloodstream | MSG51 |
| 79 | HA9 | Kw176-9/13 | 25-09-13 | Medical | Bloodstream | MSG52 |
| 80 | HB1 | Kw3101/11 | 18-10-11 | Medical | Bloodstream | MSG53 |
| 81 | HB2 | Kw81-8/12 | 07-08-12 | Medical | Bloodstream | MSG54 |
| 82 | HB3 | Kw3-10/12 | 01-10-12 | Surgical | Bloodstream | MSG55 |
| 83 | HB4 | Kw212-12/12 | 22-12-12 | Medical | Bloodstream | MSG56 |
| 84 | HB5 | Kw112-3/13 | 09-03-13 | Surgical | Bloodstream | MSG57 |
| 85 | HB6 | Kw68-4/13 | 03-04-13 | Pediatric | Bloodstream | MSG58 |
| 86 | HB7 | Kw333-6/13 | 26-06-13 | Surgical | Bloodstream | MSG59 |
| 87 | HB8 | Kw24-7/13 | 02-07-13 | Surgical | Bloodstream | MSG60 |
| 88 | HB9 | Kw189-3/14 | 07-03-14 | Medical | Bloodstream | MSG61 |
| 89 |  | ATCC22019 |  |  | Reference strain |  |

^a^Patient number MH1, MH2, MH3 etc. are for patients from the Maternity Hospital with letters ‘C’ and ‘D’ indicating colonizing strain and duplicate strain, respectively. MH60H and MH61H are isolates from health care workers from the Maternity Hospital. HA1, HA2 etc. are isolates from patients from Hospital A and HB1, HB2 etc. are for isolates from patients from Hospital B.

High-resolution fingerprinting of *Candida parapsilosis* isolates suggests persistence and transmission of infections among neonatal intensive care unit patients in Kuwait

Mohammad Asadzadeh^1^, Suhail Ahmad^1^, Noura Al-Sweih^1^, Ferry Hagen^2,3^, Jacques F. Meis^3,4^ and Ziauddin Khan^1*^

^1^Department of Microbiology, Faculty of Medicine, Kuwait University, Kuwait

^2^Department of Medical Mycology, Westerdijk Fungal Biodiversity Institute, Utrecht, Netherlands

^3^Department of Medical Microbiology and Infectious Diseases, Canisius Wilhelmina Hospital (CWZ), Nijmegen, Netherlands

^4^Centre of Expertise in Mycology, Radboudumc/CWZ, Nijmegen, Netherlands

*Corresponding author: Prof. Ziauddin Khan

**Supplementary Table 2: In vitro susceptibility of 88 *C. parapsilosis* isolates against three antifungal drugs**

| Antifungal agent | Minimum inhibitory concentration (MIC, μg/ml) | | |
| --- | --- | --- | --- |
|  | Range | MIC_50_ | MIC_90_ |
| Amphotericin B | 0.002-0.19 | 0.0125 | 0.047 |
| Fluconazole | 0.016-3 | 0.19 | 0.75 |
| Caspofungin | 0.016-0.75 | 0.25 | 0.5 |

MIC_50_ and MIC_90_, MIC required to inhibit 50% and 90% of the isolates, respectively
